# Effects of On-Court Tennis Training Combined with HIIT versus RST on Aerobic Capacity, Speed, Agility, Jumping Ability, and Internal Loads in Young Tennis Players

**DOI:** 10.5114/jhk/189691

**Published:** 2024-12-06

**Authors:** Jorge E. Morais, Bulent Kilit, Ersan Arslan, Jose A. Bragada, Yusuf Soylu, Daniel A. Marinho

**Affiliations:** 1 Department of Sport Sciences, Instituto Politécnico de Bragança, Bragança, Portugal.; 2 Research Centre for Active Living and Wellbeing (LiveWell), Instituto Politécnico de Bragança, Bragança, Portugal.; 3 Faculty of Sport Sciences, Tokat Gaziosmanpasa University, Tokat, Türkiye; 4 Department of Sport Sciences, University of Beira Interior, Covilhã, Portugal.; 5 Research Centre in Sports, Health and Human Development (CIDESD), Covilhã, Portugal.

**Keywords:** change of direction, racket sports, combined training, technical ability, perceived exertion

## Abstract

The purpose of this study was to compare the effects of on-court tennis training (OTT) combined with high-intensity interval training (HIIT) or repeated sprint training (RST) on the physiological, kinematic, kinetic, and perceptual responses of young tennis players. Twenty-four male tennis players (age 13.6 ± 0.3 years) were randomly assigned to either the OTT + HIIT group (n = 12) or the OTT + RST group (n = 12) three times per week for six weeks. Both groups trained for the same total training time with passive rest in each session. A number of physiological, performance and perceptual responses were measured before and after the 6-week training intervention. All variables showed a significant improvement over time, with maximal oxygen uptake showing the greatest improvement (p < 0.001, η^2^ = 0.97). The 5-m sprint (p = 0.044, η^2^ = 0.17), repeated sprint ability (p = 0.021, η^2^ = 0.22), and T-drill agility (p = 0.048, η^2^ = 0.17) showed a significant group effect. The OTT + RST group had a lower internal training load (better scores), a lower rate of perceived exertion (better scores), and higher scores in the Physical Activity Enjoyment Scale (PACES) at both times compared to the OTT + HITT group. These results demonstrate that OTT + RST appears to be a more effective training approach to improve speed and agility-based performance responses with more enjoyment in young tennis players.

## Introduction

In addition to improving the technical and tactical aspects of the game, the development of young tennis players should promote and provide a harmonious physical development and the improvement of various motor skills (Söğüt, 2016). Therefore, it is common to focus mainly on these two dimensions: (i) improving physical fitness, and; (ii) training of technical skills applied in the context of the game (on-court drills). Regarding the usefulness and effectiveness of on-court tennis training (OTT), it seems to be evident that it has a significant effect on tennis performance (Fernandez-Fernandez et al., 2017; Kilit and Arslan, 2019). However, it seems correct to assume that mixed training consisting of an OTT component and a component specifically aimed at improving physical fitness may be even more fruitful (Harrison et al., 2015; Ouertatani et al., 2022).

High-intensity interval training (HIIT) and repeated sprint training (RST) are two methods widely used in sports training (Bishop et al., 2011; Engel et al., 2018). Although they have different characteristics, these two popular training methods involve the repetition of high-intensity exercise followed by recovery periods. This means that, for HIIT, longer recovery intervals than those used in RST are interspersed with high-intensity exercise sessions. Exercise intervals in HIIT can range from 20 s to several minutes, depending on the protocol (Laursen and Jenkins, 2002). HIIT aims to improve both aerobic and anaerobic capacity, providing a broader range of cardiovascular benefits (Ortiz et al., 2024). On the other hand, RST focuses on repeated sprints of short duration and maximal intensity, usually 5 to 10 s, followed by short periods of recovery (≤ 60 s), with the goal of developing explosive abilities and anaerobic endurance (Bishop et al., 2011) which are needed in tennis. This method is specific to sports that require short and intense bursts of effort, such as tennis. The core of RST is to develop the ability to accelerate quickly, change direction, and recover after repeated intense efforts (Kyles et al., 2023). This method has specific demands that are very similar to those of playing tennis. In fact, the specificity of training is a critical factor in improving athletic performance (McArdle et al., 2010). Both methods have their advantages and can be incorporated into tennis training depending on the individual goals and needs of each player.

The literature reports findings on the use of HIIT (Durmuş et al., 2023) and RST (Brechbuhl et al., 2018) in tennis. However, although both forms seem to be effective in improving the skills of tennis players, there is little evidence to compare these training methods, especially in young players. To the best of our knowledge, only Fernandez-Fernandez and co-workers (2012) compared these two methods. Those authors reported that both methods promoted similar improvements in aerobic fitness. However, when compared to HIIT, RST improved repeated sprint ability to a greater extent, while HIIT promoted improvements in tennis-specific endurance (Fernandez-Fernandez et al., 2012). Therefore, by comparing both methods and using tests or drills that are commonly used in a tennis match as a main outcome, coaches can gain deeper insight into which training method can improve tennis performance or is more appropriate in each training period. In addition to the technical and physical aspects of training, it can be argued that different training approaches may have unique effects on players' perceptions of their enjoyment, which in turn may increase or decrease their motivation for their activity (Weiss et al., 2001). This means that there are training programs that can be more enjoyable for players. In this situation, coaches can have a significant impact on youth sports programs by designing or implementing training programs that are both physically and technically efficient and that increase players’ motivation and enjoyment.

In this context, the aim of this study was to compare the effects of OTT combined with HIIT or RST on aerobic capacity, speed, agility, jumping ability, and internal loads in young tennis players. It was hypothesized that both complementary training methods would promote meaningful improvements in all variables. However, RST (as a complement to OTT) would be more effective in improving these responses.

## Methods

### Participants

Twenty-four young male tennis players (age: 13.6 ± 0.3 years) were divided into two combined training groups, either the OTT + HIIT group (n = 12, age: 13.6 ± 0.2 years, body height: 162.0 ± 8.8 cm, body mass: 54.2 ± 8.9 kg; maturity offset: −0.2 ± 0.3 years; peak height velocity: 13.8 ± 0.2 years) or the OTT + RST group (n = 12, age: 13.6 ± 0.3 years, body height: 161.1 ± 8.4 cm, body mass: 51.9 ± 7.9 kg; maturity offset: −0.3 ± 0.3 years; peak height velocity: 13.9 ± 0.3 years) and classified as Tier 3 athletes (McKay et al., 2022). All were right-handed tennis players with at least two years of experience in tennis training and competition. Players were randomly assigned to one of the training groups. Afterwards, a previous group comparison was carried out to avoid mismatches in the measured variables (Fernandez-Fernandez et al., 2017). At the beginning of the intervention, there were no significant differences between the groups. Before the study began, players and their parents were informed in detail about the procedures and voluntary written consent was obtained. The study was carried out in accordance with the Declaration of Helsinki and approved by the Tokat Gaziosmanpasa University’s Ethics Committee (approval code: E-47940-14-01-03; approval date: 30 June 2021).

### Research Design

Two groups of young tennis players were used to compare the responses to a set of psychophysiological and performance tests after a 6-week training program. Two combined training protocols (OTT + HIIT vs. OTT + RST) were compared with similar total training time per session (approximately 60 to 80 min). The intervention consisted of one week of baseline testing (pre-test), six weeks of training, and one week of final testing (post-test). To prevent the negative effects of mental and physiological exhaustion, athletes participated in three training sessions per week, separated by at least two days during the six-week training period. In addition, players played a weekend match during the training intervention. The study took place during the preparation period of the summer competition season (from February to March, 2023). All tests were carried out simultaneously (from 16:00 to 20:00 h) in the same order (players and tests) on an indoor hard court. The relative humidity (40–45%) and temperature (15–20°C) of the air remained constant throughout the investigation. Details of the training intervention can be found in the supplementary file (S1).

### Anthropometrics and Maturity Offset

On the first day, body mass (kg) was measured using a bioelectric impedance analyzer (BC-418, Tanita, Tokyo). A stadiometer (Holtain Ltd., UK) was used to measure participants’ sitting and standing heights (cm). Players’ maturity was measured as reported by others (Mirwald et al., 2002). The first step was to calculate the maturity offset, which represents the years predicted before or after peak height velocity (PHV, years). This calculation was done using the following equation: Maturity offset = −9.236 + 0.0002708 (leg length × sitting height) – 0.001663 (age × leg length) + 0.007216 (age × sitting height) + 0.02292 (body mass/height × 100). The maturity offset value was then subtracted from the players’ chronological age to estimate the PHV.

### Physical Fitness

The Hit and Turn tennis test (HTTT) was used to estimate maximal oxygen uptake (VO_2max_). After a standard 5-min warm-up that included leaping, low-intensity running and dynamic stretching, each player completed the HTTT to assess their level of tennis-specific aerobic fitness. The Tennis-Specific Endurance Test is an on-court acoustically controlled, progressive fitness test for tennis players. The HTTT was administered according to the methods reported by other authors (Ferrauti et al., 2011). In this test, each player’s HR (bpm) was continuously measured and recorded using HR monitors (Polar V800, Polar Inc., Finland). The highest HR value during the test was recorded as HR_max_. The maximal completed level was used to determine VO_2max_ (ml/kg/min). After the test, VO_2max_ was estimated as:


$$\;\;{\text{VO}}_{2\max}=33.0+\left(1.66\cdot HTTT\right)$$

where VO_2max_ was maximal oxygen uptake (ml/kg/min) and HTTT was the player’s final level in the Hit and Turn tennis test (a.u.) (Ferrauti et al., 2011).

Participants completed three trials of the countermovement jump (CMJ) test, separated by 120 s of rest. The best attempt was then used for additional analysis. Players began by standing with their feet shoulder-width apart and their hands on their hips in preparation for the CMJ. They were then instructed to counter-rotate their lower limbs (knee flexion to approximately 90°) before performing a vertical jump (Sampaio et al., 2023). They were advised to land with their lower limbs straight to avoid knee flexion and advised to land at the same starting position. A portable force plate was used to evaluate the player's performance (Newtest, Finland). The height of the jump (cm) was recorded for subsequent analysis.

The Triple-Hop distance test (THD, cm) was used as a strong indicator of lower limb strength and power. In this test, players were taught to make three consecutive hops to reach the maximum distance while maintaining their balance and avoiding hand or leg contact with the ground (Hamilton et al., 2008). For the horizontal jump, each participant performed three trials. The interval between each trial was two to three minutes of passive rest. To prevent fatigue, a passive rest period of 4 to 5 min was allowed between each jump attempt. The best attempt (i.e., the largest) was used for further analysis. A standard tape measure (RossCraft, Canada) was used to measure all jump performances.

Each athlete performed a 20-m linear sprint test (with 5-m, 10-m, and 20-m intervals, s). The starting point was 70 cm behind the first pair of photocells (Witty, Microgate, Bolzano, Italy) that marked the starting line. Participants were instructed to accelerate as fast as possible (maximum effort) until they passed the 20-m timing gate. There were four sets of photocells: the starting line, five meters, ten meters, and twenty meters. The portable wireless photocell equipment was placed at the player’s waist level. Players completed two trials with a 120-s rest interval in between. The fastest time of the two sprints was selected for further analysis.

The T-test (s) was administered to assess agility performance. The validity and reliability of this test have been demonstrated in previous research (Pauole et al., 2000). The test covers basic movements used in tennis practice and competition. The players’ task was to run from a starting position to a cone 9.14 m away, then side-step to the left without crossing their feet to another cone 4.57 m away to complete the test. They touched this cone, then side-shuffled back to the middle cone, sprinted back to the starting point, then shuffled right to a third cone that was 9.14 m away. Time was measured with the above mentioned photocell system (Witty, Microgate, Bolzano, Italy).

The repeated sprint ability (RSA) test (s) consisted of six repetitions of maximal 2 x 15-m shuttle sprints (~6 s), starting every 20 s (Buchheit et al., 2010). Players were required to remain still during the approximately 14-s rest period between each run. Players were instructed to assume their starting positions for the 20-m sprints and to wait for the start signal from a supervisor two seconds before the start of each sprint. Time was measured in seconds. The mean time of the repeated sprint test (RSA_mean_) was used as a performance indicator (Buchheit et al., 2010).

### Internal Load and Perceptual Responses

The internal training load (ITL) was calculated immediately after each session based on the players’ perceived exertion (RPE), measured using the ten-level Borg scale (Borg, 1982), and training time: ITL = RPE x time (a.u.), where the RPE was the perceived exertion (a.u.) and time was the training time (Foster et al., 2021). All participants also completed a short version of the Physical Activity EnjoymentScale (PACES) (Raedeke, 2007). This scale, which consists of eight questions rated on a 1–7 Likert scale, has been validated as a marker of physical activity enjoyment in young Turkish adolescents (Soylu et al., 2023).

### Statistical Analysis

The distribution of the data was assessed using the Shapiro-Wilk test, which showed a normal distribution. All data were presented as mean ± standard deviation. A two-way repeated measures ANOVA was used to test for time effect (pre- and post-test), group effect, and their respective interactions. The significance level was set at α = 0.05. The effect size index (eta square – η^2^) was calculated and interpreted as: (i) no effect if 0 < η^2^ < 0.04, (ii) minimal if 0.04 < η^2^ < 0.25, (iii) moderate if 0.25 < η^2^ < 0.64, and (iv) strong if η^2^ > 0.64 (Ferguson, 2009).

The inter-individual variability of all variables in each training program (i.e., OTT + HIIT and OTT + RST) was quantified using the coefficient of variation (CV%). The relative percentage (%) and Cohen’s *d* (used as an effect size indicator) were also calculated for each training program between the pre- and post-test. Cohen’s *d* values were considered trivial (< 0.20), small (0.20–0.59), moderate (0.6–1.19), large (1.2–1.99), and very large (≥ 2.0) (Hopkins et al., 2009). All statistical analyses were performed with SPSS version 26.0 (SPSS, version 26.0 for Windows; SPSS Inc., Chicago, IL, United States).

## Results

Table 1 shows the time and group effects and their respective interactions for all measured variables. All variables showed a significant improvement over time, with VO_2max_ showing the greatest improvement with a strong effect size (F = 740.215, *p* < 0.001, η^2^ = 0.97). Although there was a non-significant group effect, VO_2max_ was the only variable that increased significantly in the OTT + HIIT group. Regarding the group effect, only the 5-m sprint (F = 4.559, *p* = 0.044, η^2^ = 0.17), RSA_mean_ (F = 6.215, *p* = 0.021, η^2^ = 0.22), and T-drill agility (F = 4.394, *p* = 0.048, η^2^ = 0.17) showed differences between groups (Table 1). However, the THD, 5-m sprint, 10-m sprint, 20-m sprint, RSA_mean_, and T-drill showed a significant time x group interaction. This indicates that changing the group (from OTT + HIIT to OTT + RST) significantly increases the rate of improvement in these variables.

**Table 1:** Two-way ANOVA showing time and group effects and their interaction for the measured variables.

	Time effect		Group effect		Time x Group interaction	
	F-ratio (*p*-value)	η^2^	F-ratio (*p*-value)	η^2^	F-ratio (*p-*value)	η^2^
VO_2max_ [ml/kg/min]	740.215 (<0.001)	0.97	0.003 (0.960)	0.00	4.062 (0.056)	0.01
CMJ [cm]	74.539 (<0.001)	0.77	0.407 (0.530)	0.02	0.331 (0.571)	0.00
THD [cm]	413.778 (<0.001)	0.94	2.007 (0.171)	0.08	5.496 (0.028)	0.01
5-m Sprint [s]	537.920 (<0.001)	0.84	4.559 (0.044)	0.17	87.120 (<0.001)	0.13
10-m Sprint [s]	319.860 (<0.001)	0.91	0.521 (0.478)	0.02	8.961 (0.007)	0.02
20-m Sprint [s]	310.532 (<0.001)	0.92	0.115 (0.738)	0.01	5.953 (0.023)	0.02
RSA_mean_ [s]	292.768 (<0.001)	0.77	6.215 (0.021)	0.22	64.193 (<0.001)	0.17
T-drill agility [s]	545.409 (<0.001)	0.86	4.394 (0.048)	0.17	65.161 (<0.001)	0.10
ITL [a.u.]	3609.102 (<0.001)	0.99	54.103 (<0.001)	0.71	0.627 (0.437)	0.00
RPE [a.u.]	42.220 (<0.001)	0.64	45.852 (<0.001)	0.63	2.053 (0.166)	0.03
PACES [a.u.]	55.913 (<0.001)	0.69	184.079 (<0.001)	0.89	3.697 (0.068)	0.05

VO_2max_: maximal oxygen uptake; CMJ: counter-movement jump; THD: triple hop for distance; RSA_mean:_ mean time of repeated sprint ability test; ITL: internal training load; RPE: rate of perceived exertion; PACES: physical activity enjoyment scale; η^2^: eta squared (effect size index)

As for the ITL, both groups promoted a significant time effect with a strong effect size with an increase over the six weeks of training. There was also a significant group effect with the OTT + RST group showing a greater increase. Regarding the RPE and PACES variables, the OTT + RST group showed better PACES scores and a lower RPE at both assessment times. Both training groups showed a significant time effect with a strong effect size and with an increase between scoring moments.

**Table 2:** Descriptive data (mean ± standard deviation) of all measured variables by training program (OTT: on-court tennis training; HIIT: high intensity interval training; RST: repeated sprint training). The coefficient of variation (CV, in %) in each measurement (i.e., pre- and post-test in both training programs), the relative difference (Δ, in %) between the pre- and post-test in both training programs and Cohen’s *d* (as an effect size index) are also presented.

OTT + HIIT											
		Pre-test	CV		Post-test		CV		Δ		*d*
VO_2max_ [ml/kg/min]		45.21 ± 2.06	4.56		48.83 ± 2.29		4.69		8.0		1.66
CMJ [cm]		27.17 ± 2.37	8.72		29.21 ± 2.27		7.77		7.7		0.87
THD [cm]		368.17 ± 14.12	3.84		389.92 ± 14.52		3.72		5.9		1.51
5-m Sprint [s]		1.15 ± 0.04	3.80		1.11 ± 0.04		3.77		−3.6		0.99
10-m Sprint [s]		2.17 ± 0.10	4.76		2.09 ± 0.08		4.07		−3.7		0.88
20-m Sprint [s]		3.64 ± 0.21	5.71		3.50 ± 0.21		5.86		−3.9		0.66
RST_mean_ [s]		6.59 ± 0.10	1.45		6.47 ± 0.10		1.56		−1.7		1.20
T-drill agility [s]		12.59 ± 0.17	1.32		12.38 ± 0.18		1.44		−1.6		1.19
ITL [a.u.]		158.9 ± 9.4	5.91		377.7 ± 15.8		4.18		138.4		16.83
RPE [a.u.]		8.8 ± 0.5	5.68		9.4 ± 0.4		4.25		7.3		1.32
PACES [a.u.]		32.0 ± 1.5	4.68		35.7 ± 1.1		3.08		11.7		2.81
OTT + RST											
	Pre-test			CV		Post-test	CV	Δ		*d*	
VO_2max_ [ml/kg/min]	45.50 ± 1.85			4.06		48.63 ± 2.01	4.14	6.9		1.62	
CMJ [cm]	26.50 ± 1.98			7.46		28.83 ± 1.70	5.88	9.1		1.26	
THD [cm]	374.33 ± 17.95			4.80		401.75 ± 16.47	4.10	7.4		1.59	
5-m Sprint [s]	1.14 ± 0.05			4.05		1.05 ± 0.04	3.75	−8.4		1.98	
10-m Sprint [s]	2.16 ± 0.09			4.33		2.04 ± 0.08	3.76	−5.2		1.40	
20-m Sprint [s]	3.63 ± 0.21			5.76		3.45 ± 0.20	5.85	−5.1		0.87	
RST_mean_ [s]	6.57 ± 0.14			2.12		6.26 ± 0.13	2.02	−4.7		2.29	
T-drill agility [s]	12.55 ± 0.18			1.46		12.13 ± 0.17	1.40	−3.4		2.39	
ITL [a.u.]	128.2 ± 10.4			8.11		341.3 ± 19.0	5.56	167.6		13.91	
RPE [a.u.]	7.5 ± 0.6			8.00		8.5 ± 0.5	5.88	13.7		1.81	
PACES [a.u.]	38.9 ± 1.4			3.59		41.1 ± 1.8	4.37	5.6		1.36	

VO_2max_: maximal oxygen uptake; CMJ: counter-movement jump; THD: triple hop for distance; RSA_mean_: mean time of repeated sprint ability test; ITL: internal training load; RPE: rating of perceived exertion; PACES: physical activity enjoyment scale

**Figure 1:**
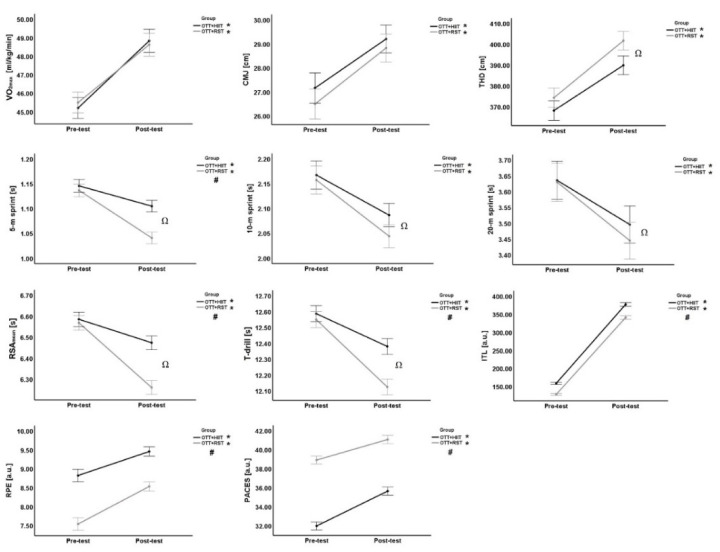
Graphic presentation of the pre- and post-test comparison for all measured variables for both groups. * significant time effect; ^#^ significant group effect; Ω: significant time x group interaction. VO_2max_: maximal oxygen uptake; CMJ: counter-movement jump; THD: triple hop for distance; RSA_mean_: mean time of repeated sprint ability test; ITL: internal training load; RPE: rate of perceived exertion; PACES: physical activity enjoyment scale

Figure 1 shows the change in all variables between the pre- and post-test. Overall, significant changes were observed in all variables, with effect sizes ranging from moderate to very large in both groups. However, it was noted that the OTT + RST group showed greater improvements than the OTT + HIIT group in 5-m, 10-m, 20-m sprint time, RSA_mean_, and T-drill agility (Figure 1). For more information, see Table 2 for the relative change between pre- and post-test and Cohen’s *d* effect sizes for each group independently. Among the physical variables, VO_2max_ was the one that presented the greatest improvement in both groups. As for the perceptual responses, PACES scores presented the greatest improvement in the OTT + HIIT group. However, it should be emphasized that this scale had better scores in the OTT + RST group at both pre- and post-test. Thus, despite a smaller relative difference and effect size noted in the OTT + RST group, this group showed better scores on the PACES (Table 2).

## Discussion

The aim of this study was to compare the effects of on-court tennis training (OTT) combined with high-intensity interval training (HIIT) or repeated sprint training (RST) on aerobic capacity, speed, agility, jumping ability, and internal loads in young tennis players. The main findings indicate that both groups significantly improved their physiological and performance responses and promoted an increase in the ITL. However, the OTT + RST group tended to show greater improvements compared to the OTT + HIIT group and with greater enjoyment (i.e., greater PACES and lower RPE scores).

The literature reports the effects of complementary training in addition to “standard” training in several sports (Harrison et al., 2015; Milanović et al., 2015). Overall, the results of studies on this topic show that all these training programs improve physical performance of players, regardless of the sport, and should therefore be used as a supplement to the “standard” programs. In the specific case of tennis, as an intermittent sport, HIIT and RST are the most studied training programs, both of which seem to elicit the physiological profile of the players (Brechbuhl et al., 2018; Fernandez-Fernandez et al., 2017). However, depending on the specificity of the training program, some characteristics can be meaningfully improved. For example, it was shown that HIIT training programs allowed for the improvement of players’ technical abilities regardless of age and the competition level (Durmuş et al., 2023). On the other hand, it was shown that RST programs might be more effective in improving the repeated sprint ability, a specific quality needed in tennis players (Fernandez-Fernandez et al., 2012). Thus, it can be argued that both training programs elicit the physiological profile of players, but depending on the characteristics that coaches aim to elicit, one or another may be more appropriate.

The present data showed that both training programs tended to elicit significant physiological and performance responses in players and to increase their ITL. Nevertheless, of all the variables analyzed, VO_2max_ was the only one that improved more in the OTT + HIIT group compared to the OTT + RST group (but without a significant group effect). Indeed, it has been suggested that HIIT programs induce aerobic fitness and endurance in tennis players (Durmuş et al., 2023; Fernandez-Fernandez et al., 2012). It was suggested that training programs that elicit VO_2max_ may help increase the players’ rate of recovery, which can have a meaningful impact on their performance during the game (Morais et al., 2023). Notwithstanding, the OTT + HIIT group also significantly improved all remaining physiological and performance variables involving specific technical skills or drills. This is consistent with what others have previously found (Durmuş et al., 2023).

Conversely, the OTT + RST group showed a significant time x group interaction in the sprints, THD, RSA_mean_, and T-drill variables. In tennis, the ability to change direction quickly and move quickly over short distances are key performance factors (Chapman and Sheppard, 2011; Parsons and Jones, 1998). Consequently, the current results showed that the RST program elicited more specific characteristics. To our knowledge, only one study has analyzed the effects of these two training programs on tennis players (Fernandez-Fernandez et al., 2012). That study was conducted on adult players, thus there is no information in the literature about such effects on youth players. Fernandez-Fernandez et al. (2012) obtained similar results since both training programs significantly improved players’ VO_2max_, yet the HIIT program promoted greater effects on the players’ overall endurance. Conversely, the RST program was more effective in improving players’ repeated sprint ability, which is strongly related to the intrinsic characteristics of a tennis match. The RST programs were also tested to understand their effect under hypoxic conditions (Brechbuhl et al., 2020). It has been shown that RST programs under hypoxic conditions elicit physiological and technical responses in specific tennis tests, such as the tennis-specific test of exhaustion and repeated sprint ability (Brechbuhl et al., 2020). Therefore, at least in tennis, RST programs are more likely to promote meaningful effects on specific tennis tests that are strongly related to performance.

Another important issue in youth sports is related to the enjoyment that should or must be part of training of youth players. For several decades, evidence on this topic has indicated that enjoyment of practice is considered a key factor for motivated behavior and permanence in youth sports (McCarthy et al., 2008). The comparison between the two methods (OTT + HIIT vs. OTT + RST) showed that the OTT + RST method improved the physiological and performance responses with a lower ITL compared to the OTT + HIIT group. This was achieved with a smaller RPE and greater PACES scores at both evaluation moments. This suggests that players had less perceived exertion and more enjoyment while performing the OTT + RST program. Indeed, the literature suggests that training programs designed for young athletes should be balanced in terms of training loads, recovery, and enjoyment from a holistic perspective (Faigenbaum, 2009; Weiss et al., 2001). Specifically in youth tennis, it has been highlighted that enjoyment is a key factor in determining the motivation of young players to maintain their participation in the sport (Weiss et al., 2001). The processes of overtraining and under recovery can lead to limitations that can play a critical role and negatively affect the development of youth athletes in general (Pelka and Kellmann, 2017). In the specific case of young tennis players, it has been shown that players who had a higher training volume were more likely to experience burnout symptoms (Mouelhi-Guizani et al., 2022). Therefore, it can be argued that it is the responsibility of coaches to design and implement training programs that are effective in improving players’ physical fitness and technical skills, while at the same time being enjoyable for athletes. In addition, current data suggest that it is possible to achieve similar or greater improvements with a lower internal training load, which may be a key factor in reducing the likelihood of burnout.

The main strengths of the present research are that there is no one-size-fits-all training approach for improving tennis performance in youth players. Both training programs were able to promote meaningful improvements in aerobic capacity, speed, agility, jumping ability, and internal loads in young tennis players. However, in terms of practicality, the OTT + RST program proved to be a more time-efficient strategy that improved aerobic adaptations, tennis-specific technique, and endurance with lower training volume requirements compared to OTT + HIIT. Additionally, the results indicate that OTT + RST results in higher levels of enjoyment and lower perceived exertion compared to OTT + HIIT. Therefore, the OTT + RST intervention is a more practical approach to improving speed, agility-based performance outcomes, and enjoyment in young tennis players. Nevertheless, it can be argued that a combination of both methods in a well-rounded training program may be the most effective approach to meet the diverse physical demands of tennis play. The main limitations are that these findings are only applicable to this age group and male players. Since there is a paucity of information on this topic, future studies should focus on understanding the effects of such training programs (and others) on tennis players of different age groups of both sexes. Researchers and coaches should also evaluate and monitor other variables or tests that may have a strong relationship with tennis performance.

## Conclusions

This study suggests that both training programs improved aerobic and anaerobic power and technical skills in young tennis players. However, the OTT + RST program showed more meaningful improvements with larger effect sizes in short sprints, repeated sprint ability, and change of direction ability than the OTT + HIIT program. The OTT + RST group also had lower internal training loads, lower perceived exertion, and higher enjoyment scores than the OTT + HIIT group. Therefore, coaches and practitioners should be aware that, at least for 13 to 14 year old tennis players, the OTT + RST program may be more appropriate. That is, for similar or greater improvements compared to the OTT + HIIT program, the internal training load is lower and the enjoyment is greater.

## Figures and Tables

**Table S1:** Training program and features of each session for six weeks of combined training.

			OTT + HIIT			OTT + RST		
Weeks		OTT		HIIT		OTT		RST
1	2 (3 x 30-s Star / 30-s rest), 60-s rest between sets			2 (3 x 30-s Sprint / 30-s rest) 60-s rest between sets	2 (3 x 30-s Star / 30-s rest), 60-s rest between sets		2 (4 x 20-m (10 + 10-m) all-out, 25-s rest), 3-min rest between sets	
	2 (4 x 30-s Box / 30-s rest), 60-s rest between sets			2 (4 x 30-s Sprint / 30-s rest) 60-s rest between sets	2 (4 x 30-s Box / 30-s rest), 60-s rest between sets		2 (5 x 20-m (10 + 10-m) all-out, 25-s rest), 3-min rest between sets	
	2 (5 x 30-s Suicide / 30-s rest), 60-s rest between sets			2 (5 x 30-s Sprint / 30-s rest) 60-s rest between sets	2 (5 x 30-s Suicide / 30-s rest), 60-s rest between sets		2 (6 x 20-m (10 + 10-m) all-out, 25-s rest), 3-min rest between sets	
2	2 (4 x 30-s Box / 30-s rest), 60-s rest between sets			2 (4 x 30-s Sprint / 30-s rest) 60-s rest between sets	2 (4 x 30-s Box / 30-s rest), 60-s rest between sets		2 (5 x 21-m (7 + 7 + 7-m) all-out, 25-s rest), 3-min rest between sets	
	2 (5 x 30-s Suicide / 30-s rest), 60-s rest between sets			2 (5 x 30-s Sprint / 30-s rest) 60-s rest between sets	2 (5 x 30-s Suicide / 30-s rest), 60-s rest between sets		3 (4 x 21-m (7 + 7 + 7-m) all-out, 25-s rest), 3-min rest between sets	
	2 (6 x 30-s Star / 30-s rest), 60-s rest between sets			2 (6 x 30-s Sprint / 30-s rest) 60-s rest between sets	2 (6 x 30-s Star / 30-s rest), 60-s rest between sets		3 (5 x 21-m (7 + 7 + 7-m) all-out, 25-s rest), 3-min rest between sets	
3	2 (5 x 30-s Suicide / 30-s rest), 60-s rest between sets			2 (5 x 30-s Sprint / 30-s rest) 60-s rest between sets	2 (5 x 30-s Suicide / 30-s rest), 60-s rest between sets		3 (4 x 20-m (5 + 5 + 5 + 5-m) all-out, 25-s rest), 3-min rest between sets	
	2 (6 x 30-s Star / 30-s rest), 60-s rest between sets			2 (6 x 30-s Sprint / 30-s rest), 60-s rest between sets	2 (6 x 30-s Star / 30-s rest), 60-s rest between sets		3 (5 x 20-m (5 + 5 + 5 + 5-m) all-out, 25-s rest), 3-min rest between sets	
	2 (7 x 30-s Box / 30-s rest), 60-s rest between sets			2 (7 x 30-s Sprint / 30-s rest), 60-s rest between sets	2 (7 x 30-s Box / 30-s rest), 60-s rest between sets		3 (6 x 20-m (5 + 5 + 5 + 5-m) all-out, 25-s rest), 3-min rest between sets	
4	2 (3 x 60-s Star / 60-s rest), 90-s rest between sets			2 (3 x 60-s Sprint / 60-s rest), 90-s rest between sets	2 (3 x 60-s Star / 60-s rest), 90-s rest between sets		3 (5 x 20-m (10 + 10-m) all-out, 25-s rest), 3-min rest between sets	
	2 (3 x 60-s Box / 60-s rest), 90-s rest between sets			2 (3 x 60-s Sprint / 60-s rest), 90-s rest between sets	2 (3 x 60-s Box / 60-s rest), 90-s rest between sets		3 (5 x 20-m (10 + 10-m) all-out, 25-s rest), 3-min rest between sets	
	2 (4 x 60-s Suicide / 60-s rest), 90-s rest between sets			2 (4 x 60-s Sprint / 60-s rest), 90-s rest between sets	2 (4 x 60-s Suicide / 60-s rest), 90-s rest between sets		3 (6 x 20-m (10 + 10-m) all-out, 25-s rest), 3-min rest between sets	
5	2 (3 x 60-s Box / 60-s rest), 90-s rest between sets			2 (3 x 60-s Sprint / 60-s rest), 90-s rest between sets	2 (3 x 60-s Box / 60-s rest), 90-s rest between sets		3 (5 x 21-m (7 + 7 + 7-m) all-out, 25-s rest), 3-min rest between sets	
	2 (4 x 60-s Suicide / 60-s rest), 90-s rest between sets			2 (4 x 60-s Sprint / 60-s rest), 90-s rest between sets		2 (4 x 60-s Suicide / 60-s rest), 90-s rest between sets	3 (6 x 21-m (7 + 7 + 7-m) all-out, 25-s rest), 3-min rest between sets	
	2 (4 x 60-s Star / 60-s rest), 90-s rest between sets			2 (4 x 60-s Sprint / 60-s rest), 90-s rest between sets		2 (4 x 60-s Star / 60-s rest), 90-s rest between sets	4 (5 x 21-m (7 + 7 + 7-m) all-out, 25-s rest), 3-min rest between sets	
6	2 (4 x 60-s Suicide / 60-s rest), 90-s rest between sets			2 (4 x 60-s Sprint / 60-s rest), 90-s rest between sets		2 (4 x 60-s Suicide / 60-s rest), 90-s rest between sets	3 (6 x 20-m (5 + 5 + 5 + 5-m) all-out, 25-s rest), 3-min rest between sets	
	2 (5 x 60-s Star / 60-s rest), 90-s rest between sets			2 (5 x 60-s Sprint / 60-s rest), 90-s rest between sets		2 (5 x 60-s Star / 60-s rest), 90-s rest between sets	4 (6 x 20-m (5 + 5 + 5 + 5-m) all-out, 25-s rest), 3-min rest between sets	
	2 (5 x 60-s Box / 60-s rest), 90-s rest between sets			2 (5 x 60-s Sprint / 60-s rest), 90-s rest between sets		2 (5 x 60-s Box / 60-s rest), 90-s rest between sets	4 (6 x 20-m (5 + 5 + 5 + 5-m) all-out, 25-s rest), 3-min rest between sets	

OTT: on-court tennis training; HIIT: high-intensity interval training; RST: repeated sprint training.

**Figure S1:**
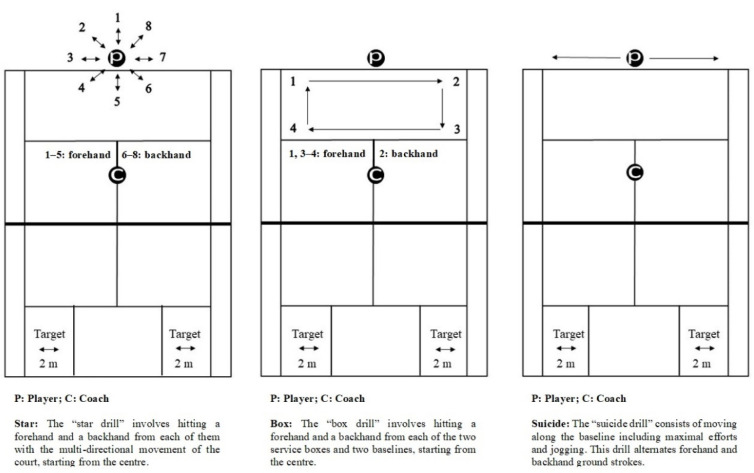
Description of on-court tennis training drills.

## Supplementary file S1

### Training Intervention

The OTT+HIIT and OTT+RST groups followed a similar training program regarding training volume during the 6-week intervention (Table S1). After a 10-min standardized warm-up, players performed 20 min of tennis-specific activities, which involved on-court hitting against an opponent, focusing on consistency, and targeting specific areas of the court (e.g., cross or line shots, serve-return, groundstrokes, volleys). The main part of the training session lasted for 20 to 40 min and included OTT followed by either running-based HIIT or RST. There was a recovery period of 2 to 3 min between the OTT and HIIT or RST sessions. OTT aimed to improve starting speed, acceleration, and speed endurance during tennis strokes, particularly the transition from forehand to backhand. After completing the main training components, a 10-min cool-down period involved stretching exercises. There was a minimum of 48 h between each training session to prevent injuries and minimize fatigue.

The training program incorporated maximal and submaximal running intensities, placing high demands on stroke quality. The primary objective was to direct all shots to specific target areas on the baseline. Various on-court tennis drills were performed in the OTT sessions using a racket and a ball (Figure S1). These drills followed procedures adapted from previous studies, ensuring a structured approach to training. During the OTT sessions, a coach positioned opposite service boxes that fed players’ balls. The coach ensured that balls were delivered at consistent frequencies and velocities. Experienced coaches, who had a Turkey Tennis Federation level 3–4 certification and 10 to 15 years of experience, supervised the OTT sessions. The drills were conducted on three tennis courts, with two players on each court. The specific drill structure consisted of 2–3 sets with 5–6 repetitions, each lasting 30 to 60 s of work (10 to 20 strokes). There was a rest period of 30 to 60 s between repetitions and a rest period of 60 to 90 s between sets. The frequency of ball feeding was approximately one ball every 3 s. Heart rate (HR) measurements were continuously monitored using HR monitors to assess the intensity of the drills. HIIT and RST sessions were individual sessions involving interval training without a racket and lasted approximately 8 to 20 min (Figure S1). During the HIIT sessions, all players achieved a heart rate (HR) exceeding 85% of their maximal HR. The structure of the HIIT session involved 2–3 sets with 4 to 5 repetitions, each lasting 30 to 60 s of work. Between each set, there was a rest period of 30 to 60 s, and between sets, a longer rest period of 90 s was applied. The rest periods were passive, allowing for recovery. HIIT sessions took place on a 400-m athletics track. In addition, there were training sessions dedicated to RST, which involved a combination of acceleration and deceleration running over short distances. RST sessions comprised 3–4 sets of 4–6 sprints covering approximately 20 m each. These sprints included forward running and side-to-side shuttles, incorporating either 1 or 3 direction changes (COD). During RST sessions, each sprint was followed by a 25-s active recovery period. Between sets, there was a rest period of 3 min to allow for adequate recovery. RST sessions were conducted on a tennis court surface, providing a specific training environment for players. All participants received verbal encouragement throughout each session to enhance their motivation and performance.
